# Comment on Billant et al. p53, A Victim of the Prion Fashion. *Cancers* 2021, *13*, 269

**DOI:** 10.3390/cancers15010309

**Published:** 2023-01-03

**Authors:** Susan W. Liebman, Irina L. Derkatch, Sangeun Park, Sei-Kyoung Park

**Affiliations:** Department of Pharmacology, University of Nevada, Reno, NV 89557, USA

The p53 tumor suppressor is a central protein in the fight against cancer. Mutations that inactivate at least one p53 allele occur in more than half of all cancer cells. Finding p53 nuclear and cytoplasmic amyloid inclusions in many tumor cell lines and cancer biopsies suggests, but does not prove, that amyloid formation inactivates p53 and that conformationally-converted p53 can be transmitted in a prion-like fashion (reviewed in [1,2]). We recently read the article by Billant et al. [3], which argues against evidence that the tumor suppressor, p53, can exist as a prion. While we agree proof that p53 forms a prion in mammalian cells and tissues is lacking, we affirm that p53 can form a stable prion in yeast.

While the initial prion hypothesis addressed the unusual features of the mammalian PrP protein, much of what we know about prions comes from studies in yeast, where prions control several endogenous traits [4,5,6]. Prions are altered, usually amyloid-based, aggregated protein conformations that drive the conversion of normally folded protein molecules with the same primary structure into their prion conformation. To continue growing and to allow the transfer/infection of these conformations into other cells, prion aggregates sheer to produce small prion seeds.

Concerning p53 prion formation in yeast, Billant et al. [3] tested and disproved the non-conventional hypothesis that certain dominant negative p53 mutants automatically and inevitably fold into the prion conformation, and that this is the sole explanation for their dominant negative effect. However, it is important to note that this hypothesis has no parallels in prion research. Indeed, even for hereditary prion diseases, such as Gerstmann–Straussler–Scheinker Disease [7], it takes years for the mutant PrP protein to take on a prion conformation.

A limited number of proteins can form prions in living cells. Even for these proteins, the de novo appearance of prion seeds in the absence of infection is rare. While certain mutations or the overexpression of the protein or its prion domain can enhance the de novo appearance of prion seeds, the frequency of new seed formation is low. However, once a protein forms a prion, it is fairly stable.

We showed that either wild-type or p53-R175H mutant human p53 expressed in yeast can indeed propagate stably in either a bona fide prion or non-prion form [8]. Furthermore, the formation of the p53 prion inactivates the wild-type p53’s functional activity as a transcription factor measured in yeast by color or growth assays. The “p53, a Victim of Prion Fashion” review incorrectly implies that we only showed prion formation for tagged p53. In fact, we showed that both untagged and tagged wild-type p53 can form and propagate a prion (see [8] Figures 1F and 4).

Billant et al. [3] showed that the transient overexpression of dominant negative p53-R175H or p53-R248Q does not convert wild-type p53 into a prion in the entire culture. Rather, in these cultures, mutant p53 inhibits co-expressed wild-type p53, likely because the mutant and wild-type molecules are incorporated into tetramers together, resulting in inactive tetramers. The findings that turning off the expression of the mutant p53 protein allows the wild-type p53 to regain full function, and that the dominant negative effect of the mutant is reduced proportionally by increasing the relative level of wild-type p53, are compatible with the tetramer poisoning hypothesis, but not with the prion hypothesis.

However, we emphasize that Billant et al.’s finding that mutant p53 does not inevitably take on a prion form or always exist as a prion does not lead to the interpretation that mutant or wild-type p53 cannot sometimes propagate as prions. Indeed, by definition, prion-forming proteins can take on and stably exist in either non-prion or prion forms.

In yeast, the SUP35 protein spontaneously forms the [*PSI*^+^] prion in the presence of the [*PIN*^+^] prion at a rate of ~7 × 10^−7^ [9,10]. The overexpression of SUP35, whether attached to GFP or not, greatly enhances this frequency [11,12]. However, RNQ1-GFP overexpression does not significantly promote the formation of its prion form, [*PIN*^+^] [13].

Although we have not quantified the frequency with which p53 forms a prion in yeast, we easily found p53 prions following transient p53-YFP overexpression in cells constitutively expressing untagged functional p53. We screened for cells that lost p53 activity using a color assay. When we turned p53-YFP synthesis back on in cells that retained their p53 activity, p53-YFP was diffused in the cell nuclei. This is what we see when we express p53-YFP in yeast lacking the p53 prion. However, in many of the isolates that lost p53 activity, when we turned the p53-YFP synthesis back on, p53-YFP was in nuclear and cytoplasmic aggregates, indicative of prion formation. Furthermore, cells with inactive p53 only appeared following p53-YFP overexpression, but not in the control samples expressing an empty vector. We characterized one such isolate extensively showing that the prion phenotypes of p53 inactivation and aggregation could be transferred to non-prion cells by infecting them with prion cells’ cytoplasm via either cytoduction or transfection.

Importantly, we showed that the prion was maintained if just untagged p53 synthesis was retained. Indeed, p53 aggregates could be seen in prion cells lacking p53-YFP expression by staining them with the amyloid-binding dye thioflavin T (see [8] Figure 1F). However, the transient interruption of both p53-YFP and untagged p53 synthesis caused permanent loss of the prion (see [8] Figure 4).

Furthermore, we showed that tagged p53-175H-YFP can also exist in either the non-prion form, with diffuse fluorescence in the nuclei, or in the prion form with frequent cytoplasmic fluorescent foci. We obtained prion cultures of p53-175H-YFP by infecting non-prion p53-175H-YFP cultures with untagged wild-type p53 prions. Upon terminating the wild-type prion protein expression, p53-175H-YFP retained the prion/cytoplasmic aggregate phenotype.

In sum, untagged p53 can form a stable prion in yeast, exhibiting all of the defining characteristics of a yeast prion, including its infrequent de novo appearance. Recognizing that p53 can form a prion and that this causes a loss of p53 function is critical when considering therapeutic treatments for cancers associated with p53 inactivation. This is because, in cases where a p53 prion is the cause of p53 inactivation, the addition of p53 may be a poor therapeutic approach as the p53 prion seed should attract and inactivate the added p53. It is also important to learn what conditions promote p53 to form a prion and what p53 mutations promote this conversion. Surprisingly, new research shows that the inactivation of p53 has a silver lining. Specifically, in the presence of neurodegeneration caused by C9orf72poly(PR), p53 inactivation is therapeutic [14]. Thus, there could be a physiological benefit to p53 prion formation, explaining its intrinsic propensity to aggregate [2].

In conclusion, rather than being a victim of prion fashion, p53 is a sterling example of the likely relevance of prion formation to human health.
